# Nociceptin is present in synovial fluid of patients undergoing total knee arthroplasty

**DOI:** 10.1186/s13018-020-01789-1

**Published:** 2020-07-16

**Authors:** Thomas A. Verbeek, Nancy Ruth Jarbadan, Charles Davis, Julia Caldwell

**Affiliations:** 1 Department of Anesthesiology & Perioperative Medicine, H187, Penn State Health Milton S. Hershey Medical Center, 500 University Dr., Hershey, PA 17033 USA; 2 Department of Anesthesiology & Perioperative Medicine, Penn State College of Medicine, Hershey, PA USA; 3 Department of Orthopedics and Rehabilitation, Penn State Bone and Joint Institute, Penn State Health Milton S. Hershey Medical Center, Hershey, PA USA; 4 Vitality Pain Clinic, Bowling Green, KY USA

**Keywords:** Nociceptin, Orphanin FQ, Osteoarthritis, Neuroinflammation, Arthroplasty

## Abstract

**Background:**

Osteoarthritis is a mechanical abnormality characterized by chronic joint pain associated with degeneration of the articular cartilage, synovitis, and local inflammation, leading to loss of function and pain. A connection exists between the peripheral nervous system and inflammatory joint degeneration. The process by which inflammation is influenced by the nervous system is known as neuroinflammation. One of the neuropeptides involved in peripheral neuroinflammation is nociceptin, a peptide related to the opioid class of substances. Nociceptin has both pro- and anti-inflammatory effects. Some studies show that nociceptin can be measured in synovial fluid, while other studies have not been able to detect it. The presence of nociceptin in synovial fluid could imply a molecular role for the neuropeptide in the joint, both physiologically as well as pathophysiologically. The goal of this pilot study was to determine whether nociceptin was present in the synovial fluid of osteoarthritic knees.

**Methods:**

Patients undergoing primary total knee arthroplasty were enrolled after Institutional Review Board approval was obtained. Synovial fluid was aspirated from patients’ operative knee joints and blood samples were obtained. A commercially available enzyme Immunoassay kit was used to test for nociceptin. A linear mixed-effects model was developed to account for the repeated measurements and baseline covariates. Least squares (adjusted) means were derived from the model to compare the sample types and to compare subgroups.

**Results:**

Twenty patients were included in this study. Nociceptin was detected in the synovial fluid and plasma of all patients. The mean concentration (± standard deviation) of nociceptin in synovial fluid was 28.7 ± 18.2 pg/ml. The mean concentration of nociceptin in plasma was 45.2 ± 24.3 pg/ml pre-procedure, and 40.1 ± 20.6 pg/ml post-tourniquet deflation. The nociceptin concentration in synovial fluid was significantly lower than the nociceptin concentration in plasma, both pre-procedure and post-tourniquet deflation (*p* = 0.002 and *p* = 0.016 respectively). The nociceptin concentration in both plasma and synovial fluid was significantly lower in females versus males (*p* = 0.012).

**Conclusion:**

We demonstrated that nociceptin is present in synovial fluid and plasma of patients undergoing total knee arthroplasty. This implies a potential role for nociceptin in modulating inflammation in osteoarthritis.

**Trial registration:**

ClinicalTrials.gov, NCT02528916. Retrospectively registered on August 19, 2015,

## Background

Osteoarthritis (OA) is a mechanical abnormality characterized by chronic joint pain associated with degeneration of the articular cartilage, synovitis, and local inflammation [1], leading to loss of function and pain. This condition affects 10% of men and 18% of women over the age of 60 worldwide and places a large economic burden on society [2, 3]. Treatment is directed towards pain management, with joint replacement for end-stage disease [4]. Currently, diagnosis relies on patients reporting symptoms combined with radiological imaging. Unfortunately, symptoms only develop once the disease is irreversible [4]. This has led to the search for biochemical markers to detect early disease, before irreversible damage occurs. None have been validated clinically [5]. Because of this lack of validated biomarkers, very little progress has been made in the discovery of disease-modifying agents for OA.

It is well known that a connection exists between the peripheral nervous system and inflammatory joint degeneration [6]. The process by which inflammation is influenced by the nervous system is known as neuroinflammation [7]. This process is mediated by the peripheral release of sensory neuropeptides from nerve terminals. One of the neuropeptides involved in peripheral neuroinflammation is nociceptin. This neuropeptide has both pro- and anti-inflammatory effects [8, 9]. Nociceptin is an endogenous nociceptin opioid receptor (NOP) agonist with similar structure to endogenous opioids [10]. It is, however, different from classic opioids like morphine, in that it lacks agonistic activity at classical opioid receptors (mu, kappa, and delta) [11, 12] and resists naloxone-induced antagonism [13, 14]. Some studies show that nociceptin can be measured in synovial fluid [11], while other studies have not been able to detect it [15]. The presence of nociceptin in synovial fluid could imply a molecular role for the neuropeptide in the joint, both physiologically as well as pathophysiologically. Clarifying the role of nociceptin in joints may prove useful in the diagnosis, as well as the treatment of OA, especially with the advent of the opioid analgesic cebranopadol. This opioid is unique in its mechanism of action as it acts not only as an agonist on mu, kappa, and delta opioid receptors, but also as an agonist on the nociceptin receptor [16].

The goal of this pilot study was to determine whether nociceptin was present in the synovial fluid of osteoarthritic knees. These data will be used to design a study comparing healthy subjects’ synovial fluid and plasma nociceptin concentrations to the same measurements in patients with osteoarthritis.

## Methods

The study was registered retrospectively with ClinicalTrials.gov (registration number NCT02528916). EQUATOR guidelines for a cross-sectional observational study were followed. Twenty-two patients were enrolled between May and November 2014. This was a convenience sample of patients aged 18–80 years, diagnosed with osteoarthritis, undergoing primary total knee arthroplasty (TKA). Patients who were not hemodynamically stable, had undergone prior joint arthroplasty at the current surgical site, or had active infection of their knee joint, were excluded from the study. The same orthopedic surgeon performed all of the procedures. Anesthetic technique consisted of general anesthesia combined with regional anesthesia (femoral nerve block, subarachnoid block, or lumbar epidural). Standard institutional anesthetic technique was followed.

Two blood samples (3 ml each from the antecubital vein) and a synovial fluid sample (5 ml) were obtained from each patient. The first blood sample was collected preoperatively, during intravenous catheter placement. After induction of anesthesia, a tourniquet was applied to the operative thigh to limit blood loss and to ensure a dry operative field. The synovial fluid sample was aspirated from the knee joint after skin incision, and the second blood sample was collected 5 min after the thigh tourniquet was deflated. Blood samples were collected in BD Vacutainer K2 EDTA Plus Blood Collection Tubes (BD, Franklin Lakes, NJ, USA) and placed on ice until processed by the addition of the protease inhibitor aprotinin and centrifugation (1600×*g*, 4 °C, 15 min). The plasma supernatant was aliquoted into RNAse/DNAse-free tubes and stored at − 80 °C until assayed. Synovial fluid was collected, aliquoted, and stored at − 80 °C until assayed. The concentration of nociceptin in plasma and synovial fluid was quantified by fluorescent enzyme immunoassay using the nociceptin/orphanin FQ enzyme immunoassay kit from Phoenix Pharmaceuticals, Burlingame, CA, USA.

Additional patient information, including patient age, gender, and body mass index was collected. Patient-reported pre- and postoperative pain was assessed using an eleven-point numerical rating scale (NRS) [17]. Preoperative pain was assessed on admission. Post-procedure pain was assessed as the first pain NRS score provided by the patient in the post-anesthesia recovery unit. The patient’s status according to the American Society of Anesthesiologists (ASA) Physical Status Classification System was recorded.

Study data were collected and managed using REDCap (Research Electronic Data Capture) electronic data capture tools hosted at Penn State Health Milton S. Hershey Medical Center [18]. Data were analyzed using the SAS® software, version 9.4 (SAS Institute Inc., Cary, NC, USA). A linear mixed-effects model was developed to account for the repeated measurements and baseline covariates. Least squares (adjusted) means were derived from the model to compare the sample types and to compare subgroups, such as males versus females. *p* values less than 0.05 were considered statistically significant.

## Results

Twenty-two patients with clinically diagnosed osteoarthritis were enrolled. Technical reasons prevented us from obtaining synovial fluid samples from two patients—they were excluded from the analysis. Demographic data for the patient population are shown in Table 1.

**Table 1:** Patient demographic data

	Male	Female	Total
*n*	8	12	20
Age (years)	61 ± 7	66 ± 7	64 ± 8
Weight (kg)	105 ± 21	87 ± 18	94 ± 21
Body mass index (kg/m^2^)	33 ± 7	33 ± 8	33 ± 7
ASA status	2.4 ± 0.7	2.7 ± 0.5	2.6 ± 0.6

Data are presented as mean ± standard deviation, or counts (*n*)

Sixteen patients received general anesthesia plus ultrasound-guided femoral nerve blocks with a long-acting local anesthetic (7 males, 9 females). Three patients underwent the procedure with continuous lumbar epidural anesthesia combined with general anesthesia (2 females and 1 male). These three participants underwent bilateral total knee replacements. In these patients, synovial fluid samples were obtained from the first joint operated on only, and plasma samples were obtained after release of the first tourniquet. One female patient received subarachnoid anesthesia combined with general anesthesia, and had a femoral nerve block after the procedure. Nociceptin was detected in all plasma and synovial fluid samples (Table 2).

**Table 2:** Nociceptin is present in synovial fluid and plasma

	Pre-procedure (plasma)	Post-tourniquet release (plasma)	Synovial fluid
Nociceptin concentration (pg/ml)^*^	45.2 ± 24.3	40.1 ± 20.6	28.7 ± 18.2
Range (pg/ml)	7.5 to 120.7	9.0 to 104.0	5.2 to 123.6

^*^Data are presented as mean ± standard deviation

The nociceptin concentration in synovial fluid (28.7 ± 18.2 pg/ml) was significantly lower than the nociceptin concentrations in plasma, both pre-procedure (*p* = 0.002) and post-tourniquet deflation (*p* = 0.016). The difference in nociceptin plasma concentrations pre-procedure and post-tourniquet deflation was not statistically significant (*p* = 0.34).

There was no significant correlation between nociceptin levels and age (*p* = 0.12), body mass index (*p* = 0.23), preoperative pain scores (*p* = 0.53), or postoperative pain scores (*p* = 0.36). However, there was a significant correlation between nociceptin levels and gender (*p* = 0.012). The nociceptin concentration in plasma and synovial fluid was significantly higher in males than in females (Table 3).

**Table 3:** Nociceptin concentrations in males versus females

Sample	Male nociceptin concentration (pg/ml)^*^	Female nociceptin concentration (pg/ml)^*^	*p* value
Pre-procedure (plasma)	60.5 ± 16.0	47.5 ± 18.6	0.035
Post-tourniquet release (plasma)	55.7 ± 14.3	42.2 ± 16.7	0.016
Synovial fluid	48.3 ± 20.7	26.0 ± 9.4	0.0008

^*^Data are presented as mean ± standard deviation

## Discussion

We demonstrated measurable levels of nociceptin in both the plasma and synovial fluid of osteoarthritis patients undergoing knee arthroplasty. The presence of this neuropeptide in synovial fluid suggests a role for this molecule either in the physiology of the joint or the pathophysiology of OA. This may have diagnostic as well as therapeutic implications for the diagnosis and management of OA, including disease modification.

The detection of nociceptin in synovial fluid in our study is qualitatively similar to the results of Fiset et al. [11], although quantitatively we found nociceptin concentrations that were orders of magnitude lower. We detected nociceptin in plasma, while Fiset et al. did not detect nociceptin in autologous plasma samples. We used freshly harvested patient samples that were immediately processed and then analyzed within a few days to a few months, whereas Fiset et al. used banked synovial fluid and plasma samples. These differences may have contributed to the discrepancies noted. Our results for nociceptin concentrations in plasma are consistent with plasma nociceptin concentrations of 9.6 ± 2.6 pg/ml and 10.7 ± 5.6 pg/ml reported by Ertsey et al. and Ko et al., respectively, for healthy controls [19, 20]. Our results also contrast with the report of Kumar et al. [15], where nociceptin was not detected in synovial fluid; however, our processing and assay methods differed from those used in Kumar’s study.

There was no control group. Our goal with this study was to determine if nociceptin was present or absent in synovial fluid, making a control group unnecessary. We did not obtain a synovial fluid sample from subjects’ contralateral knees as this was deemed an unacceptable risk to the patient.

Given the exploratory nature of this pilot study consisting of only 20 subjects, one cannot place undue significance on results obtained when analyzing subgroups. However, the *p* value of 0.0008, for the difference in synovial fluid nociceptin concentration between males and females, suggests a difference that warrants further investigation. Abundant evidence indicates greater pain sensitivity among females compared with males [21]. Holtzman et al. reported that women were more likely to report severe pain and disability when presenting for total hip replacement surgery than their male counterparts [22]. Srikanth et al. showed that females tended to have more severe osteoarthritis in the knee compared to males [23]. More research is needed to clarify this possible association between lower levels of nociceptin in synovial fluid and increased osteoarthritis in females.

In conclusion, nociceptin is present in the synovial fluid of patients undergoing primary knee arthroplasty. This implies a potential role for nociceptin in modulating inflammation in osteoarthritis.

## Data Availability

The datasets generated and analyzed during the current study are available from the corresponding author on reasonable request.

## Abbreviations

ASA: American Society of Anesthesiologists
NOP: Nociceptin opioid receptor
NRS: Numerical rating scale
OA: Osteoarthritis
REDCap: Research Electronic Data Capture
TKA: Total knee arthroplasty
